# Structural variation in the chicken genome identified by paired-end next-generation DNA sequencing of reduced representation libraries

**DOI:** 10.1186/1471-2164-12-94

**Published:** 2011-02-03

**Authors:** Hindrik HD Kerstens, Richard PMA Crooijmans, Bert W Dibbits, Addie Vereijken, Ron Okimoto, Martien AM Groenen

**Affiliations:** 1 Animal Breeding and Genomics Center, Wageningen University, Marijkeweg 40, 6709 PG, Wageningen, the Netherlands; 2 Research and Technology Centre, Hendrix Genetics, P.O. Box 30, 5830 AE, Boxmeer, The Netherlands; 3 Cobb-Vantress Inc, P.O. Box 1030, Siloam Springs, AR 72761, USA

## Abstract

**Background:**

Variation within individual genomes ranges from single nucleotide polymorphisms (SNPs) to kilobase, and even megabase, sized structural variants (SVs), such as deletions, insertions, inversions, and more complex rearrangements. Although much is known about the extent of SVs in humans and mice, species in which they exert significant effects on phenotypes, very little is known about the extent of SVs in the 2.5-times smaller and less repetitive genome of the chicken.

**Results:**

We identified hundreds of shared and divergent SVs in four commercial chicken lines relative to the reference chicken genome. The majority of SVs were found in intronic and intergenic regions, and we also found SVs in the coding regions. To identify the SVs, we combined high-throughput short read paired-end sequencing of genomic reduced representation libraries (RRLs) of pooled samples from 25 individuals and computational mapping of DNA sequences from a reference genome.

**Conclusion:**

We provide a first glimpse of the high abundance of small structural genomic variations in the chicken. Extrapolating our results, we estimate that there are thousands of rearrangements in the chicken genome, the majority of which are located in non-coding regions. We observed that structural variation contributes to genetic differentiation among current domesticated chicken breeds and the Red Jungle Fowl. We expect that, because of their high abundance, SVs might explain phenotypic differences and play a role in the evolution of the chicken genome. Finally, our study exemplifies an efficient and cost-effective approach for identifying structural variation in sequenced genomes.

## Background

Structural variation within the genome, including insertions, duplications, deletions, and inversions of up to multiple kilobase pairs, have recently been described in a variety of species, including humans [1-3], mice [4], rats [5], silkworms [6] drosophila [7], and dogs [8]. These genomic variations were recently found to be widespread, encompassing 5% of the human genome [9], and are thought to be involved in (co)determining complex phenotypes [10,11].

The contribution of structural variants (SVs) to complex phenotypes has been measured by association analyses of variance in gene expression levels (traits) and the presence of SVs. SNPs and SVs have been shown to account for 83.6% and 17.7%, respectively, of the total detected genetic variation in gene expression, with only a limited overlap [12]. The effect that SVs have on gene expression is likely underestimated given the much less completeness and accuracy with which SVs could be queried at that time. In humans, SVs have been associated with sporadic and Mendelian diseases, such as Williams-Beuren syndrome, mental retardation, and red-green color blindness. SVs have also been associated with complex human traits, such as autism, schizophrenia, Crohn's disease, and susceptibility to HIV infection [13]. Because of their association with human diseases, the importance of SVs has become increasingly apparent [9,14,15]. For most other species, including the major farm animals, chickens, cattle, and pigs, the extent and biological consequences of SVs have remained largely unknown due to the lack of a cost-effective approach for detecting SVs.

Until recently, comparative genomic hybridization (array-CGH) was the most commonly used method for detecting SVs [16]. Fosmid paired-end sequencing, which is a more laborious technique, has been used to detect SVs larger than 8 kb [17,18]. The inability to resolve smaller SVs using array-CGH results in the over-representation of larger SVs in current databases of structural variation (e.g., http://projects.tcag.ca/variation/). The resolution of array-CGH, though extremely costly, can be improved by using high-resolution whole-genome tiling arrays. Most of these SVs have been identified by methods that do not resolve SV end points at the base pair level. In addition, methods like array-CGH are based on a reference genome that currently does not encompass all SVs within the population and, thus, is limited in scope. Genomic regions that are the result of deletions not present in the reference genome are not captured by the array and not analyzed for SVs.

Next generation sequencing (NGS) technology was recently shown to be a powerful alternative to array-CGH for identifying genomic structural variation [1,7,19]. Using paired-end sequencing, SVs can be identified with single base pair resolution. Moreover paired-end sequencing allows for the detection of balanced rearrangements in which there is no gain or loss of a genomic region, such as inversions and translocations, which cannot be identified by array-CGH. Paired-end sequencing and mapping (PEM) involves sequencing the paired ends of fragments of known insert size from a genomic DNA library and computationally mapping DNA reads to a reference genome.

Here, we used PEM on reduced representation libraries (RRLs) of pooled chicken DNA samples. In the chicken genome, only 43 (larger) SVs have been described thus far [20]. These SVs encompass 16 chicken-turkey inter-specific copy number variants (CNV) and 32 chicken-duck inter-specific CNVs, of which five CNVs overlap with inter-specific chicken-turkey CNVs [21]. In chicken, some phenotypes have already been linked to structural variation, including the pea-comb [22] and late feathering [23] phenotypes. With PEM of an RRL, we provide a cost-effective approach for exploring the presence of SVs at high resolution within four chicken breeds.

## Results

### Paired-end sequencing and mapping

To identify genomic rearrangements in the chicken genome, we applied massively parallel sequencing using the Illumina Genome Analyzer platform to sequence both ends of the genomic DNA fragments derived from the RRLs. We used pooled samples from 25 individuals to construct *Alu*I RRLs for a white egg layer line, brown egg layer line, and two different broiler lines. For the white and brown egg layer lines, the 150-200 bp *Alu*I fragments were used for creating the RRL; for the two broiler lines, 125-200 bp *Alu*I fragments were used. From the brown and white egg layer RRLs, we obtained 31.61 million and 29.70 million raw reads, respectively, and from broiler 1 and broiler 2 we obtained a total of 34.8 million and 32.4 million raw reads, respectively. Reads were filtered for the presence of the restriction enzyme tag and trimmed to 32 bases. We required a phred quality score [24] of at least 20 (Table 1) for each base in the 32-bp read. The fraction of read pairs for which both reads mapped back to the reference chicken genome (Red Jungle Fowl built WASHUC2) was 78% for broiler 1 and 77% for broiler 2 (Table 1). In the layers, the fraction was 76% (brown egg layer) and 73% (white egg layer). In all breeds the were approximately hundred thousand paired reads (0.5-0.6%)of which only one read mapped back to the reference genome, whereas up to 26% of the read pairs had no end uniquely mapping back to the reference genome.

**Table 1:** Sequencing and mapping results for the four chicken breeds analyzed for structural variation

Sequencing			Mapping						
Breed	Raw reads	**Paired l32q20**^**1**^	**Concordant**^**2 **^**%**	**Neither end**^**3 **^**%**	**One end**^**4 **^**%**	**Diff chr**^**5 **^**%**	**Too short**^**6**^	**Too long**^**7**^	**Relative orientation**^**8**^
**Brown egg layer**	31.61	23.59	76.14	23.22	0.52	0.02	470	22547	549
**White egg layer**	29.70	21.84	73.30	25.81	0.64	0.14	1019	22058	1872
**Broiler 1**	34.82	24.83	78.26	21.14	0.48	0.01	2108	21209	335
**Broiler 2**	32.28	20.64	76.60	22.64	0.54	0.07	7388	22058	1030

Paired-end sequencing of RRLs resulted in the indicated number of raw reads per breed. Sequencing read counts are in millions. Mapping percentages are relative to Paired l32q20.

^1^Paired l32q20 = paired reads had the RRL restriction tag trimmed to 32 bp and were filtered for a minimum per base quality of 20;

^2^Concordant = both reads of a read pair mapped to the expected orientation relative to each other and in the expected distance according to the RRL size range;

^3^Neither end = none of the reads of a read pair mapped to the reference;

^4^One end = only one read of a read pair was mapped;

^5^Diff chr = both reads of a read pair mapped, but to different chromosomes;

^6^Too short = both reads of a read pair mapped to the expected orientation relative to each other but at a closer distance than expected based on the RRL size range;

^7^Too long = both reads of a read pair mapped at a larger distance from each other than expected;

^8^Relative orientation = reads of a read pair mapped in another orientation relative to each other than expected based on the reference chicken genome.

To calculate the sequence coverage of the RRL, we estimated the number of fragments in the RRL by performing an *in silico Alu*I digest of the chicken genome build WASHUC2, which resulted in 583,826 fragments of 150-200 bp, whereas 947,538 fragments of 125-200 bp were obtained. We calculated RRL sequence coverage based on the paired-end reads that passed our sequence quality filters. Coverage of the RRLs ranged from 11-13X in broiler lines to 18-20X in the layer lines, indicating that we analyzed, on average, 22-40% of the haplotypes of the 25 individuals used for constructing the RRL (Table 2).

**Table 2:** RRL construction simulated by an *in silico Alu*I digest of the WASHUC2 build of the reference chicken genome

Line	Size-range	Number of fragments	Genome fraction	Sequenced (32 bp reads)	RRL coverage calculated
**Layers**	150-200	583826	101 Mb (8%)	18.7 Mb (1.5%)	37-40X
**Broilers**	125-200	947538	151 Mb (12%)	30.3 Mb (2.4%)	22-26X

Fragments were collected in corresponding size ranges as used in the *in vitro *RRL preparation. The total number of collected fragments and number of bases captured are indicators of what genome fraction was sampled. Based on trimmed reads, the fraction of the genome actually sequenced was calculated. The number of raw read pairs obtained (see Table 1) divided by the number of fragments is an indicator of the RRL coverage.

**Figure 1:**
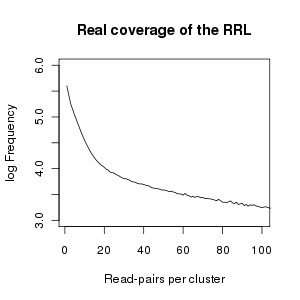
**Sequence coverage of the RRL**. On the x-axis are the obtained sequence coverages of RRL-fragments estimated by read-pair clusters and on the y-axis the frequency in which they occurred (10 log scale)

The real sequence coverage of the RRL was estimated by clustering identical paired reads and plotting the distribution of clusters according to the numbers of reads per cluster (Figure 1). The majority of the fragments in the RRL was covered by 10 paired reads.

**Figure 2:**
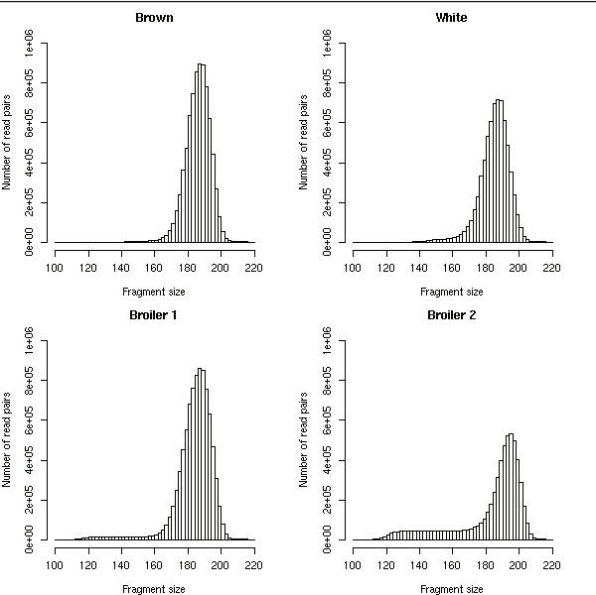
**Distribution of fragment sizes for concordantly mapping reads in the four sequenced chicken breeds**. For unclear reasons, broiler 2 had remarkably higher representation of smaller fragments (left long shoulder), whereas fragments in base pairs of the size range 180-200 were two magnitudes less abundant compared to the three other breeds.

For each breed, we calculated insert sizes for paired ends that mapped in the correct orientation (Figure 2). The results show a peak at ~185 bp and a shoulder of smaller fragments, indicating that the insert sizes were not equally distributed. The upper limit of fragment size was clearly demarcated at ~210 bp, which corresponded well to the size range of the excised fragments. Based on these results, the lower limit was estimated to be ~135 bp in the layer lines and ~110 bp in the broiler lines, which is consistent with the applied size selection. To eliminate false positives, we established size thresholds of 100 and 220 bp and considered mapping paired reads within this range as consistent with the reference genome.

### Rearrangements

In each breed, roughly 0.1% of the mapping read pairs had no concordant alignment in the reference genome, referred to as discordant paired-end reads [2,17], indicating a potential SV. Discordantly mapping read pairs are those whose distance apart is less or greater than expected from the RRL size range or in another relative orientation than expected based on the reference genome (Table 1). Paired reads that mapped to two different chromosomes (up to 0.12%) were excluded from further analysis. Discordantly mapping read pairs of the larger chicken chromosomes (1-15,20 and Z) with similar mapping coordinates and predicting a similar putative SV were clustered in 10,559 clusters. Clusters were classified as having an insert size that was too large (deletions, n = 5135), too small (insertions, n = 5241), or an incorrect orientation of ends (inversion breakpoints, n = 183) with respect to the chicken genome sequence.

Because of the high number, not all of the clusters are presumed to represent a true genomic rearrangement, but some are incorrectly mapped reads caused by sequencing errors that result in low quality mapping. Therefore, the average mapping quality of discordantly mapping read pairs was evaluated per chromosome compared to the average mapping quality scores of read pairs that mapped consistently within the reference genome. However, the average mapping quality of discordantly mapping reads was similar to the mapping quality of concordantly mapping read pairs (Table 3). We also observed that the average coverage by paired reads differed up to two-fold between chromosomes, but the number of fragments per chromosome in the RRL correlated well with chromosome size.

**Table 3:** Comparison of the mapping quality and distribution between concordantly and discordantly mapping read pairs

Chromosome	Number of mapping read pairs		Average mapping quality		Mapping density		RRL density
**1**	5329141	*15630*	67.92	*69.11*	38	*12860*	*1148*
**2**	3968343	*15049*	68.14	*71.29*	39	*10291*	*1149*
**3**	3344481	*11031*	68.87	*68.20*	34	*10303*	*1119*
**4**	2758645	*8155*	68.53	*70.40*	34	*11555*	*1098*
**5**	1975228	*5390*	68.53	*67.93*	32	*11547*	*1065*
**6**	1258393	*2782*	68.31	*69.69*	30	*13443*	*1056*
**7**	1336228	*4669*	68.78	*65.41*	29	*8221*	*1053*
**8**	1119526	*2866*	68.63	*72.82*	27	*10702*	*1067*
**9**	1016524	*3232*	68.16	*69.65*	25	*7907*	*1028*
**10**	761372	*2725*	68.20	*69.52*	30	*8278*	*1044*
**11**	677920	*1381*	68.56	*68.70*	32	*15879*	*1050*
**12**	864303	*3039*	68.33	*69.74*	24	*6758*	*989*
**13**	780565	*2107*	68.47	*66.72*	24	*8976*	*966*
**14**	740461	*3512*	67.86	*69.36*	21	*4504*	*929*
**15**	669260	*1378*	68.56	*68.47*	19	*9411*	*916*
**20**	722054	*2501*	68.78	*68.27*	19	*5592*	*911*
**Z**	1845751	*11981*	68.05	*68.79*	40	*6227*	*1271*

The number of concordant and discordant (in italics) mapping read pairs per chromosome are given. The average mapping quality of concordantly and discordantly mapping read pairs was calculated per chromosome. By calculating the mapping density, the distribution of mapping read pairs over the genome were evaluated. Mapping density was calculated by dividing the chromosome length by the number of concordantly/discordantly mapping read pairs. RRL density was calculated to ascertain the contribution of the RRL approach to differences in mapping density. RRL densities were calculated by dividing the chromosome length by the (*in silico*) estimated number of RRL fragments.

**Figure 3:**
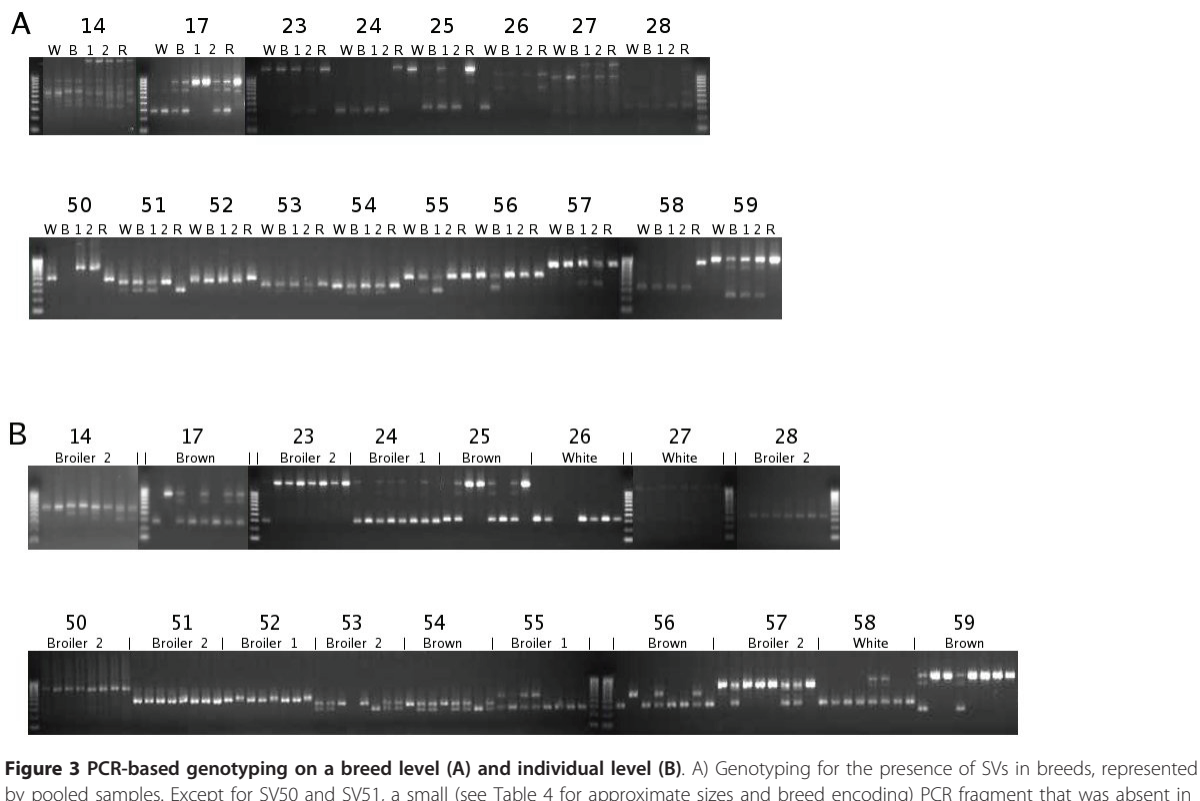
**PCR-based genotyping on a breed level (A) and individual level (B)**. A) Genotyping for the presence of SVs in breeds, represented by pooled samples. Except for SV50 and SV51, a small (see Table 4 for approximate sizes and breed encoding) PCR fragment that was absent in the reference was expected in some of the breeds that have the deletion. In SV50 and SV51, a slightly larger PCR fragment than that observed in the reference was expected in breeds that have the insertion. B) Genotyping for the presence of SVs in eight individuals of breeds in which the SV was detected in pooled samples. Except for SV50 and SV51, a small PCR fragment was expected in individuals homozygous for the deletion and SVs in which the reference genotype is too long for PCR. Heterozygous individuals in which both genotypes can be spanned (see Table 4) by PCR show two bands. In SV50 and SV51, both PCR fragments, which differ slightly in size, are expected in heterozygous individuals, whereas only the larger fragment is expected in individuals homozygous for the insertion.

To be considered as a true putative SV cluster, we required both ends to have an average mapping quality similar to concordantly mapping reads, which was ~60. In total, 7,789 clusters consisting of 3794 deletions, 3931 insertions, and 64 inversion breakpoints met this criterion. SV clusters predicting a deletion or insertion were further prioritized for confirmation screening on the basis of parameters listed in the Methods section. To validate our approach for identifying SVs, we initially evaluated 15 (SV13-28) predicted SVs (Table 4) using PCR to genotype pooled samples from the four chicken breeds with primers spanning predicted breakpoint junctions. A total of eight SVs yielded a clear PCR product of the expected size (Figure 3A). For these SVs, PCR was performed on eight individuals from breeds in which the SV was confirmed to be present by the SV-specific PCR product (Figure 3B). Individual SV-specific PCR products typed homozygous for the SV were sequenced to disentangle the rearrangement at the base-pair level. The sequence analysis results for these eight identified rearrangements were all consistent with our SV predictions.

**Table 4:** Validation structural polymorphisms

Prediction							Confirmation			
**SV**	**Span size**	**n**	**CMP**	**RE**	**aamq**	**Breed**	**Breakpoints**	**Size**	**Size in RRL**	**Breed**

**15**	251	1	X		97	2	NA			
**14**	402	3			97	1,2	10_1627991-1628223	232	170	1,2
**13**	414	2			93	W	NA			
**18**	640	1		X	99	1	NA			
**22**	661	121	X	X	77	W,B,1,2	NA			
**17**	729	4	X		94	W,2	3_110574268-110574832	564	165	W,2
**20**	780	6		X	96	W,1,2	NA			
**21**	884	1	X	X	99	1	NA			
**19**	970	2		X	99	1	NA			
**25**	1248	3			73	2	1_188914114-188915200	1086	162	B,1,2
**23**	1319	1			97	2	2_55356006-55357163	1157	162	1,2
**24**	1376	2			70	2	4_23256240-23257477	1237	139	W,B,1,2
**26**	5845	1	X		90	W	2_112569238-112574924	5686	159	W
**27**	19574	15	X		96	W,1	-	-	-	-
**28**	8128	489	X		93	2	1_61836457_61844398	7941	187	W,B,1,2
**50**	64	48			71	B,1,2	2_152470660*			1,2
**51**	86	39			69	2	3_19576932	115	201	W,B,1,2
**52**	229	141			79	B,1,2	4_43663736-43663781	45	184	W,B,1,2
**53**	274	10			76	B,1,2	6_6687386-6687469	83	191	B,1,2
**54**	283	140	X		74	B,1,2	2_46860428-46860509	81	202	B,1,2
**55**	360	4			76	1	3_67474749-67474961	212	148	1
**56**	367	21	X		72	B	1_189692870-189693048	178	189	B
**57**	544	4			69	1,2	7_28561048-28561407	359	185	12
**58**	662	2			60	1	1_44948882-44949390	508	154	W,B,1,2
**59**	868	2	X		97	2	1_99177206-99177957	751	117	B,1,2

Structural variants (SV) 13-18 were chosen before application of the empirical rule (span-size deviation) × n >500, whereas 50-59 were chosen after. Span size is the distance (in base pairs) on the reference sequence spanned by discordantly mapping read pairs. The number of observed discordantly mapping read pairs that support the presence of this structural variant is given by n. CMP is flagged in case there were also concordantly mapping read pairs observed in that particular genomic region. Discordantly mapping read pairs spanning an assembly problem in the reference genome are flagged in the RE column. The alternative mapping quality of a predicted SV is the average mapping quality calculated over discordantly mapping read pairs within a cluster. Deletion breakpoints are in the notation chr_start-stop, whereas insertion breakpoints are given in the notation chr_position. Not acquired (NA) breakpoints were due to false positive SV predictions whereas breakpoints for SV27 were not acquired for technical reasons and not accurately acquired in SV50 due to low sequence complexity. W = white egg layer; B = brown egg layer; 1 = broiler 1; 2 = broiler 2.

*Due to the low sequence complexity, the exact location of insertion could not be revealed

### Discriminating putative SVs from false positives

The results suggest that the presence of concordantly mapping reads partly overlapping the predicted SV region did not correlate with the quality of SV prediction, whereas reference errors in the predicted SV region correlated negatively. Furthermore, the results indicate that putative SVs predicted by a single or a few discordantly mapping read pairs that mapped a slightly different distance than expected were false positives, whereas the majority of putative SVs with greatly deviating mapping distances were confirmed as being true SVs. With this limited number of observations, we formulated a simple but fitting rule to determine SV clusters with a high likelihood of representing a genomic rearrangement from false positives.

**Figure 4:**
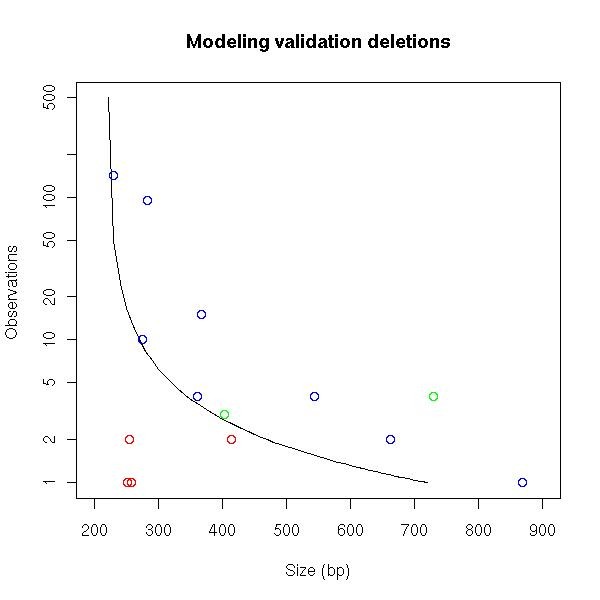
**Distinguishing putative deletions from false positives in genotyping validation results obtained by PCR**. Predicted deletions in the initial validation study that were confirmed are in green; those that could not be confirmed are in red. The black line represents the discrimination rule (span-size difference)×n >500, which is valid for 220-720 bp. The SV predictions that were selected based on the model and confirmed are in blue.

We hypothesize that the size range of targeted DNA fragments isolated from the gel might contain a very small fraction of fragments outside the established size thresholds (Figure 2). This lack of proper separation is likely caused by migration artifacts caused by secondary DNA structures. To compensate for this bias we required that SVs, predicted based on discordantly mapping read pairs that mapped to the reference between 220 and 720 bp apart, meet a representation constraint. In our proposed validation rule, we assumed an inverse relationship between the span-size deviation of a predicted SV and the number of discordantly mapping read pairs (n) required to predict a true SV. We hypothetically state that SVs meeting the abundance constraint (span-size deviation) × n >500 can be validated as true deletions. We assumed that this empirical rule is also applicable to insertions predicted by read pairs that map (too short) a distance of 32-100 base pairs. To test our empirical rule, we applied it to the subset of deletion (n = 3794) and insertion (n = 3931) clusters used in the previous validation study, obtaining 186 candidate putative deletions and two insertions. Both insertion candidates (SV50 and SV51) and a total of eight deletions (SV52-SV59), four of which narrowly met the rule constraints (Figure 4), were selected for confirmation. PCR-based genotyping analysis showed that all selected candidates were confirmed in the pooled samples (Figure 3A). We also observed that the PCR-based SV genotyping results for pools correlated well with the predicted presence of a particular SV in the breeds based on the sequence dataset (Table 4).

### Breed-specific and shared SVs

**Figure 5:**
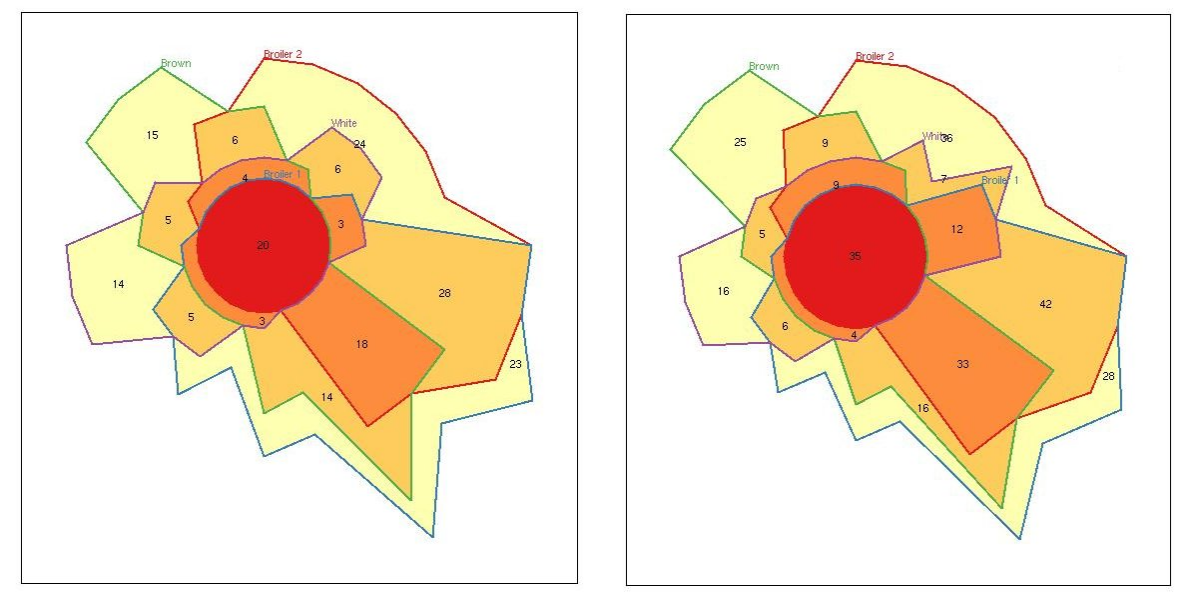
**Venn diagrams representing the distribution of predicted deletions in the four chicken breeds at mapping constraints 60 (left) and 35 (right)**. The number of structural variants is proportionally represented per breed, and line colors were assigned as follows: green = brown egg layer; blue = broiler 1; red = broiler 2; and purple = white egg layer. For example, the area that is surrounded by the blue line in the left diagram represents SVs found in broiler 1. Of these, 23 were specific for broiler 1 (yellow area), and 28 were shared with broiler 2 (dark yellow area surrounded by both the blue and red lines). The orange area surrounded by the blue, red, and green line represent 18 SVs shared by broiler 1, broiler 2, and brown egg layers. The red area in the middle of the diagram surrounded by the four line colors represents 20 SVs shared by the four breeds analyzed.

Genotyping results suggested that the presence or absence of SVs in a particular breed is fairly well predicted by the sequencing data. Therefore, we further analyzed 186 rearrangements (deletions) validated by our rule for breed specificity. We also analyzed breed specificity for 280 putative deletions that resulted from applying a less stringent read mapping quality constraint, which was also applied in previous SV detection studies [19,25]. The results were compared by plotting both data subsets in weighted Venn diagrams (Figure 5). In the validated dataset of 186 deletions, we detected the most SVs in broilers, 114 in broiler 1 and 109 in broiler 2, whereas fewer SVs were detected in the layer lines, 60 in white egg layers and 85 in brown egg layers. Ten percent of the rearrangements were present in all four breeds. SVs detected in white egg layers were 23% breed-specific, and the other 77% were evenly shared with the other breeds. The brown egg layers had the fewest breed-specific SVs (18%) and shared a remarkably high percentage (65%) with broiler 1. Broiler 1 and broiler 2 showed similar percentages of breed-specific SVs, and 36% of the SVs in broiler 2 were shared with broiler 1. Applying a less stringent mapping quality constraint resulted in a 50% increase in SVs, whereas the distribution of SVs over the four chicken breeds remained approximately the same.

### Distribution of predicted SVs

**Figure 6:**
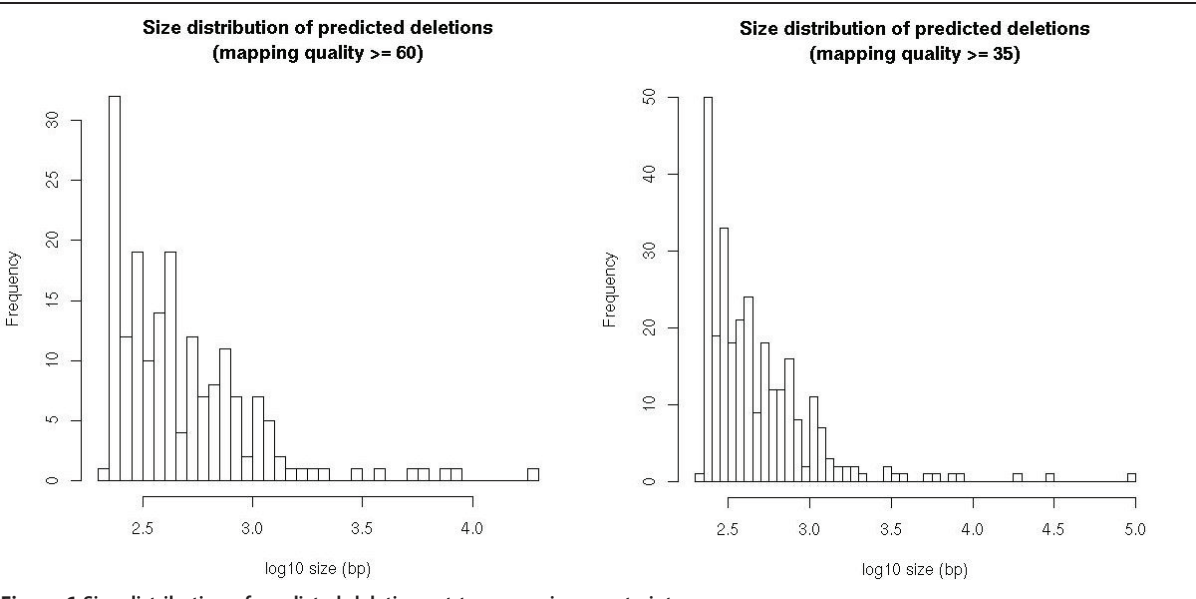
**Size distribution of predicted deletions at two mapping constraints**.

**Figure 7:**
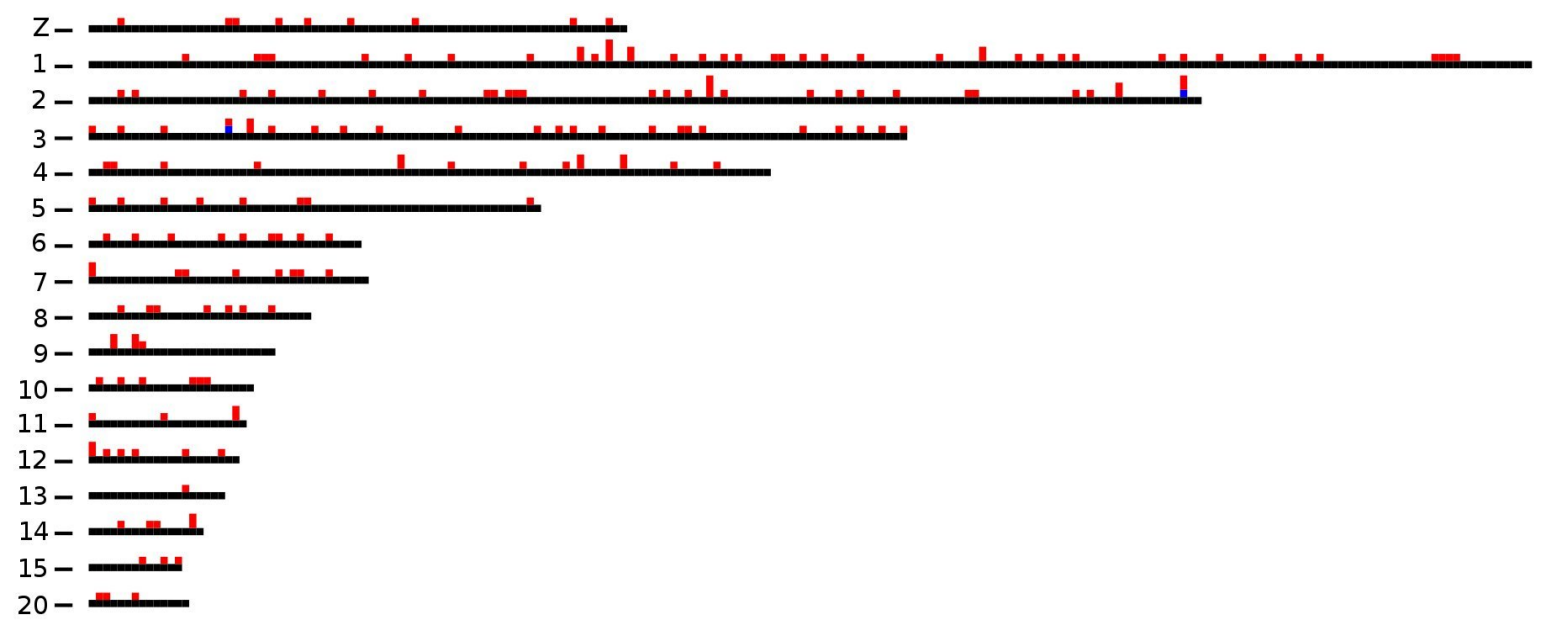
**Distribution of predicted SVs over the chicken chromosomes**. Shown are chicken chromosomes in which 186 deletions (red) and 2 insertions (blue) were identified.

The majority of detected SVs were small (Figure 6); approximately 85% of all SVs were <1 kb whereas 60% were <500 bp. However, we also predicted and validated SVs spanning multiple kilobases. Predicted SVs validated by our rule were mapped to the chicken genome, and we observed an even distribution on the chromosomes (Figure 7). Sequence annotations of the regions overlapping the identified SVs were extracted from Ensembl [26]; 44% of the SV read pairs mapped within genes. The read pairs for a minor fraction of the SVs (~2%) spanned predicted exons; these SVs were analyzed for their effects on gene annotations or gene models (Table 5). The majority of all predicted SVs represented a putative deletion of low complexity and repetitive sequence motifs in intronic or intergenic regions (Table 6). An exception is SV52, representing a deletion within gene ENSGALG00000010719, which has been annotated as DNA glycosylase FPG2.

**Table 5:** Analyses of putative deletions for their effects on gene annotations

Breakpoints*	Transcript(s)	Modification	Protein
8_4940538-4940787	ENSGALT00000005255	Truncation last exon	Flavin_mOase
14_14073018-14073274	ENSGALT00000003325	Truncation exon 9 or 5' deletion exon 10	PDZ domain
3_78504957-78505263	ENSGALT00000025445	5' deletion in last exon	Ionic channel
9_6501514-6501912	ENSGALT00000008864/40988	5' deletion in exon 4	Transcription factor
1_70753183-70753846	ENSGALT00000022933	Truncation exon 10	EGF-like
1_13962380-13963075	ENSGALT00000013428	Truncation exon 2	Unknown
11_748787-749698	ENSGALT00000002076/23151	Truncation last exon	ADP-ribosylation factor-like

Putative deletions with breakpoints predicted in exons were further analyzed in Ensembl [26]. Involved transcripts and protein functions were identified and putative modifications recorded.

*Breakpoints are estimated from the mapping results and might differ a few tens of bases from the exact genomic locations.

**Table 6:** Putative functional annotations of predicted SVs

			Coding		Repeats					
**aamq**	**n**	**% genes**	**% within exons**	**% exons**	**% CR1**^**1**^	**% GGLTR**^**2**^	**% other**^**3**^	**% TR**^**4**^	**% dust**^**5**^	**%!**^**6**^
**35**	280	43.9	0.36	5	19.6	5.3	5.0	25.0	36.1	42.9
**60**	186	43.0	0.54	3.8	18.8	4.3	3.2	26.9	36.6	41.9

SVs of data subsets aamq 35 and aamq 60 were annotated based on their mapping location on the chicken genome. SVs were analyzed to determine whether they mapped within genes, within exons, or partially overlapped exons.

^1^CR1 = chicken repeat 1 [36]

^2^GGLTR = Gallus gallus long terminal repeat

^3^other = other specific repeat classes

^4^SVs that mapped in repetitive sequences were analyzed for signatures of common repeats in the chicken genome and scanned for tandem repeats identified by Tandem Repeat Finder [37];

^5^SVs that mapped in repetitive sequences were analyzed for signatures of simple repeats identified by the DUST algorithm [38];

^6^The fraction of SVs that mapped in intronic and intergenic regions not identified as repetitive or low complexity are given in column "%!".

### SVs at base pair resolution and overlap with functional elements

**Figure 8:**
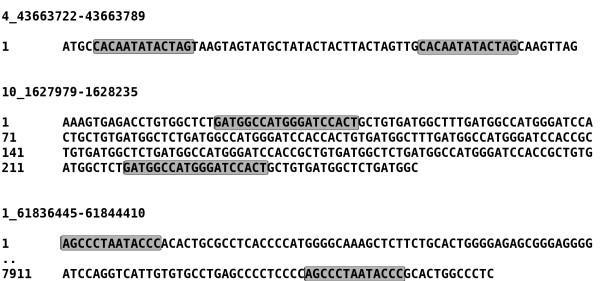
**Microhomologies detected in sequenced SVs**. Shown are the three SVs in which microhomology (grey boxes) was detected at the SV breakpoints.

All PCR-validated SVs were characterized by traditional sequence analysis to reveal their exact breakpoint locations, from which the chromosomal position and deletion/insertion sizes were derived (Table 4). Sequence losses were annotated using Ensembl [26]. For rearrangements in SV52, we analyzed the effect on the *in silico *transcript to which it was mapped. The majority of intronic deletions resulted in a loss of a variety of known repetitive motifs (Table 7). In contrast, we could not find annotations in Ensembl [26] for most losses in intergenic regions or known repeats using RepeatMasker (Smith and Green unpublished). DNA sequences at the SV breakpoints were analyzed for signatures indicating the mechanism by which the SVs formed. We identified microhomology in three sequenced SVs (Figure 8). Finally, the SV we observed in a coding region involved a deletion in the end of the last exon (ENSGALE00000116074) of transcript ENSGALT00000038211.

**Table 7:** Annotation of confirmed deletions and DNA signatures at breakpoints

Breakpoints	Gene	Exons	Repeats	Signatures
4_43663736-43663781	ENSGALG00000010719	ENSGALE00000116074		MH
2_46860428-46860509	ENSGALG00000012116			
6_6687386-6687469				
1_189692870-189693048				
3_67474749-67474961				
10_1627991-1628223	ENSGALG00000001729		trf1	MH
7_28561048-28561407	ENSGALG00000011699		dust	
1_44948882-44949390			dust	
3_110574268-110574832	ENSGALG00000016679		CR1-F0, Z-REP, trf, dust	
1_99177206-99177957				
1_188914114-188915200			dust, trf	
2_55356006-55357163	ENSGALG00000012402		dust, trf	
4_23256240-23257477	ENSGALG00000020249		dust, trf	
2_112569238-112574924			CR1-Y4, dust, trf	
1_61836457_61844398	ENSGALG00000012956		CR1-D2, Mariner1, GG, dust	MH

Deletions were annotated based on their mapping position on the chicken genome and deleted sequences were analyzed for common and more chicken-specific repeats. trf = repeats identified by Tandem Repeat Finder [37]; dust = simple repeats identified by the DUST algorithm [38]; CR1, = chicken repeat 1 [36]; Z-REP = macrosatellite family on chicken chromosome Z [39]; GG = repeats on the chicken genome identified by RECON [40]. We also analyzed the DNA sequence at SV breakpoints for signatures indicating the mechanism by which the SVs are formed, and we identified microhomology (MH) in some cases.

## Discussion

By sampling a portion of the genome from four chicken lines using stringent SV detection constraints, we detected 188 SVs encompassing ~130 kb. Assuming considerable limitation in the detection of classes of SVs by our method, the chicken genome may differ in SVs to a greater extent than in SNPs. Therefore, we counted the total number of nucleotides involved. The majority of SVs identified by our method were small deletions, most of which resulted in a loss of repetitive motifs in intronic regions or a loss of unannotated sequences in intergenic regions. Both insertions mapped to intergenic regions as sequences of a few tens of base pairs and low complexity. We also predicted rearrangements in coding regions, revealed the exact breakpoints on the reference genome for 16 SVs, and confirmed our predictions. To what extent SVs in intronic and intergenic regions contribute to the evolution of the chicken genome or chicken phenotypes remains unclear, especially because the functions of these genomic regions are largely unknown [27]. To date, studies involving the detection and exploitation of genetic variation in chicken encompass large SVs by means of CNVs but do not include smaller SVs. Our study reveals that, given their high frequency, these smaller SVs will need to be incorporated in genotyping because they might explain phenotypic differences. In addition, our data suggest that structural variation has contributed to genetic differentiation among current domesticated chicken breeds and the Red Jungle Fowl, and might have played a role in chicken genome evolution.

### RRL-based approach to SV detection

Currently, sequence-based genome-wide surveys of SVs involve the preparation of whole genome fragment libraries in combination with paired-end sequencing. Such approaches require relatively large investments, particularly if multiple individuals from multiple breeds have to be screened. This study demonstrated the potential of massive parallel paired-end sequencing of RRLs constructed from the pooled DNA of multiple individuals. SVs were predicted based on the read pair information from the paired-end sequenced small insert RRL, which was purposely created for SNP detection. The small RRL size allowed for PCR-based confirmation and characterization of the SV at the base pair level of acquired deletions and small insertions with minimal sequencing efforts. Revealing inversion and translocation breakpoints is much more laborious due to the limited information RRL approaches provide. We showed that read pair analysis of a paired-end sequenced RRL is already sufficient for obtaining a first glimpse of SVs in a particular sequenced species. This RRL based strategy put constraints on the quality of the reference genome because assembly errors will result in false positive SV predictions in reference based detection approaches. Uncertainty about the quality of assembly of some of the smaller micro-chromosomes together with computational limits at the time of this study were the reasons why we did not analyze the whole genome for SVs. An enhanced assembly of the chicken reference genome and the increasing computational power allow for improvement in the detection of SVs using our approach. Furthermore the use of multiple RRLs including large and small fragments pools, that are separately tagged and paired-end sequenced together in bulk, will considerably improve SV detection at small increase of cost. More demanding is PEM of a randomly sheared and size-selected whole genome library providing a more complete catalog of rearrangements characterized between a sample and a reference [1,19]. An even more complete picture including SVs of a larger size and more complex rearrangements will require paired-end sequencing of several libraries of different insert sizes [28]. The detection of all structural variation, which requires whole genome sequencing and *de novo *assembly, is extremely demanding. However, the identification of (small) deletions and insertions with comparable or shorter length than the standard deviation of paired-end insert sizes requires *de novo *assemblies, because such SVs cannot reliably be identified by mapping approaches. Moreover, reference-based approaches, included mapping approaches, are biased to the completeness of the reference and, thus, ignore variants in regions that are missing from the reference genome due to structural variation. Finally, *de novo *assembly has the advantage of resolving SVs to a single base pair level, and inserted sequences can be obtained [29].

### Next generation sequencing

We used a NGS approach to identify genomic rearrangements within four commercial chicken breeds by comparing their genomes to the sequenced chicken genome (Red Jungle Fowl). We excluded several classes of sequence reads from further analysis, including reads that did not show the restriction enzyme tag and those that showed more than one mismatch in the alignment. The first constraint was applied to eliminate false positive insertion predictions due to a breakdown of the RRL resulting in shorter spans of paired-end reads, whereas the second constraint was applied to reduce the number of false predictions due to sequencing errors. However, we realize that by taking these measures we also discard many read pairs because of true nucleotide variation, which occur in one of every 200 bp in the chicken [30]. The inclusion of read pairs with more than one mismatch in the alignment can be considered but has a risk of falsely predicted SVs due to mapping errors, requiring a revalidation of our proposed SV size deviation versus the observed frequency rule (Figure 4). On the other hand, reducing the mapping constraints might reveal additional true SVs potentially hidden in the considerable fraction of read pairs with only one end or no end mapped to the reference when using our mapping constraints. However, this fraction of read pairs with mapping problems might also largely represent sequences of gaps in the genome (estimated to encompass ~100 Mb in total) and, thus, cannot be mapped.

### SV distribution across breeds

Theoretically, our approach for identifying SVs allows the prediction of SVs and insight into how a predicted SV is distributed across breeds. We showed that the observed distribution of SVs is a good predictor for the actual distribution of the SV in breeds. Even with limited sampling, predicted SV distributions correlated with the PCR-based genotyping results of pooled samples (Table 3). In general, PCR-based genotyping revealed that predicted SVs are more widely shared in breeds than predicted by our sequencing-based estimation. This situation is caused by limited sampling, and the reduction of target sequence complexity by creating RRLs might have contributed to this difference. Our sampling regimen required enzyme recognition sequences flanking a SV within the size range for the RRL to include a particular SV in the RRL. Breed-specific SNPs in *Alu*I sites may have caused one or both SV alleles to not be sampled and are, thus, not predicted to be present in that breed, consequently affecting our sequencing-based estimation of SV distribution across breeds. Conversely, our PCR-based genotyping approach with pooled samples was not affected by sampling limitation or *Alu*I SNPs and revealed the presence of SVs in a breed even at allele frequencies of 0.1 (data not shown).

Because of the difference in the predicted presence of a SV in a breed and the genotyping results, we realize that the 186 SVs with which we estimated breed specificity might not be fully representative. The use of different RRL sizes (150-200 bp in layers and 125-200 in broilers) is reflected in a 1.5-2-fold difference in the SVs detected in broilers and layers. The fairly large percentage of SVs shared in broilers can be interpreted as being due to the effects of selection during line development by commercial companies and is consistent with the results of recent SNP genotyping [31], but it might be over-estimated in our study due to the difference in RRL construction. The percentage of predicted SVs shared by brown egg layers and broiler 1, however, is an indication that these breeds are more genetically related compared to the other breeds. Recent SNP genotyping results for brown and white egg layers and three broiler lines also indicated that the brown egg layer breed is more closely related to broiler lines than to white egg layers [31], which is in agreement with our conclusion based on SV distribution.

### Abundance, location, and size of SVs in the chicken genome

The reduction in the percentage of the genome covered by sequencing a RRL instead of randomly sampling the whole genome placed high constraints on the detection of SVs. The actual amount of SVs is likely much higher because we only sampled those that are flanked by restriction sites, and such that the intermediate sequence length of the variant was in the size range of the RRL. Large insertions were not expected to be detected because our RRL approach only allows for the detection of up to about 170 bp, the size between the maximum RRL fragment size (~200 bp) minus the mapping size of two completely overlapping reads (32 bp)

Although the larger SVs are most likely under-represented in our data due to the constraints of the applied detection method, we can conclude that the majority of SVs in the chicken genome are smaller than 1 kb (Figure 6). This finding is consistent with human studies [2] in which SV abundance inversely correlated with SV size. We observed that 99% of the predicted SVs were located in intronic (43%) and intergenic regions (56%), which together comprise ~90% of the chicken genome. As expected, SVs were less abundant in coding regions because, like SNPs, they are more likely to have negative impacts and be eliminated by purifying selection. Moreover the observed lower abundance of SVs in coding region is consistent with the idea that the most common rearrangement mechanism requires substrates, such as microhomology, low copy repeats, and segmental duplications, which are more abundant in non-coding regions [10,32,33]. In 3 of 15 sequenced SV breakpoints, we were able to identify signatures in the DNA sequence indicating the mechanism by which SVs are formed. All identified signatures involved microhomology at the breakpoint junction that resulted from either nonhomologous end-joining or replication fork stalling and template switching events [34]. Other SVs did not show a clear sequence signature.

## Conclusion

We provided a first glimpse of the abundance and genomic locations of structural variation in the chicken genome by identifying 188, mostly small, rearrangements, some of which were in coding regions, though a majority was located in non-coding regions. Based on the present data, we expect to find thousands of small (<1 kb) and hundreds of larger rearrangements in the whole chicken genome, encompassing more nucleotides than SNPs, and that are putatively involved in phenotypic variation. We observed that structural variation has contributed to genetic differentiation among current domesticated chicken breeds and the Red Jungle Fowl. Finally, we showed that little sequencing effort on a reduced representation of a genome is sufficient for the detection and base pair level annotation of a variety of SVs in a sequenced genome.

## Methods

### SV detection using RRLs of pooled samples and NGS

Individual DNA samples were pooled according to breed and the genome complexity reduced by isolating a fraction of a complete genome digest. The isolated reduced representation library (RRL) was paired-end sequenced using Illumina genome Analyzer technology. The paired-end reads were aligned to the reference chicken genome WASHUC2 build and SVs are identified as significant differences between the mapping distances identified by the paired-end reads and the size range used for constructing the RRLs. Deletions relative to the reference genome were identified by paired ends spanning a genomic region in the reference genome longer than the size in the RRL, whereas insertions were identified by paired ends spanning a shorter genomic region in the reference sequence than expected based on the RRL. Inversion breakpoints were detected by paired ends that mapped in a different relative orientation compared to the reference genome.

### Paired-end sequencing

Genomic DNA was extracted from 30 μl of blood from 25 unrelated F_0 _individuals from brown and white egg layer lines and two broiler lines consisting of 13 males and 12 females (Broiler 1) and 25 males (Broiler 2) using a Puregene DNA isolation kit (D-70KA; Gentra Systems, Inc., USA).

The RRLs were prepared by digesting 25 μg of pooled DNA using 1,000 units of the restriction enzyme *Alu*I in a total volume of 240 μl. The selection of the restriction enzyme was based on the 10-fold reduction of genome complexity in the optimum size range (100-200 bp) of the sequencing technology platform (Genome Analyzer, Illumina). The digested DNA sample was fractionated on a 10% precast polyacrylamide gel (Biorad) at 100 V for 3 h and stained with ethidium bromide. The size fractions were sliced out of the gel and the DNA was mechanically sheared and and eluted over night in 300 μl recovery buffer (8 mM Tris pH 8.0, 0.08 mM EDTA, 1.25 M ammonium acetate. After a 15-min incubation at 65°C, the eluent was purified using a Montage DNA Gel Extraction Device (Millipore Corporation, Bedford, MA) and precipitated with isopropanol. The DNA was washed with ethanol and re-suspended in DNA hydration solution (Gentra Systems, Inc., USA).

We prepared the Genome Analyzer paired-end flow cell according to the manufacturer's protocol.

Five picomole aliquots of the RRLs were processed using the Illumina Cluster Generation Station (Illumina, Inc., USA) following the manufacturer's recommendations. The Illumina GAII Genome Analyzer (Illumina, Inc., USA) was programmed to produce a theoretical fixed read length of 36 bp.

Images from the instrument were processed using the manufacturer's software to generate FASTQ sequence files. Paired reads that had both the RRL restriction tag and a per base phred (Ewing and Green, 1998) quality score of at least 20 were selected using custom Perl scripts and aligned to the chicken genome (WASHUC2) using the MAQ [35] algorithm v0.7.1 with parameters -1 32 -2 32 -a 220.

### Artifact removal

Alignment results were analysed according to the MAQ [35] documentation by using custom perl and bash scripts. Paired reads in which one or both ends were mapped with more than one mismatch or mapped ambiguously on the reference sequence were excluded from analysis, as these would not reliably detect SVs. Discordantly mapping read pairs in which the two ends mapped >220 bp apart were classified as deletions and subsequently clustered based on overlapping mapping positions. SVs longer than 100 kb disrupted clustering and were excluded. Read pairs that mapped within 100 bp of each other were classified as insertions, whereas read pairs that mapped with one of the two ends in the incorrect orientation were classified as inversions. Both insertions and inversions were also clustered based on mapping positions by applying custom made Perl scripts.

### Confirmation of identified SVs

For each SV cluster, we recorded the number of reads spanning the rearrangement, regardless of whether a normally mapping pair was observed or whether a sequence gap in the WASHUC2 build was present within the genomic range in which the deletion was predicted. SV clusters were prioritized for validation as follows: (i) an alternative mapping quality score of at least 60, (ii) both reads of a discordantly mapping pair mapped within a single predicted Ensembl exon or gene [26], and (iii) the genomic sequence flanking the SV allows primer design (Primer3Plus [41]) within 200 bp. We applied these criteria for selecting candidates distributed over the 220 bp-20 Kbp (deletions) and 32 bp-100 bp (insertions) size ranges. If these criteria yielded more than one candidate, the candidate with the highest alternative mapping quality score was selected.

Primers were designed to span the possible breakpoint by locating them 40-200 bp outside the mapping location of discordantly mapping read pairs. The minimum and maximum aberrant PCR product size was expected to be the sum of the minimum/maximum fragment size in the RLL and required flanking genomic region for primer development. PCR reactions were initially performed on DNA of the Red Jungle Fowl reference animal UCD001 and the pooled samples of all four breeds. For breeds in which the rearrangements were detected, individual samples were genotyped by PCR. The PCR products of homozygous individuals, or samples in which only the aberrantly sized product resulted, were sequenced on a conventional Sanger capillary sequencer and the results compared to the reference sequence using megablast with parameter -F F to identify breakpoints. Both ends of the PCR product on the reference (Red Jungle Fowl) were sequenced and mapped to the reference to ensure that it originated from the expected genomic position.

Confirmed SVs were defined as those for which PCR reactions resulted in a distinct band in the expected size range in at least the breed for which the rearrangement was predicted and with no matching band in the UCD001 reference animal. The PCR results had to be supported by unambiguous sequencing data mapping confirming the rearrangement.

## Availability and requirements

The data from this paper have been submitted to the NCBI Short Read Archive (http://www.ncbi.nlm.nih.gov/Traces/sra/sra.cgi) under accession no. SRA026771.

The SVs identified in this study that have not been confirmed and annotated at the base pair level are available upon request, awaiting a central repository of structural variation in genomes.

## Authors' contributions

HHDK designed and developed the SV prediction method and wrote the manuscript. BWD and RPMAC prepared the samples and performed the initial validation and genotyping analysis. AV and RO selected the animals to be sequenced and collected the samples. MAMG and RPMAC coordinated and supervised experiment implementation and assisted in the preparation of the manuscript. All authors read and approved the final manuscript.
